# Programmable nanophotonic planar resonator filter-absorber based on phase-change InSbTe

**DOI:** 10.1038/s41598-023-40269-4

**Published:** 2023-08-14

**Authors:** Israel Alves Oliveira, I. L. Gomes de Souza, V. F. Rodriguez-Esquerre

**Affiliations:** 1 Graduate School of Electrical Engineering, Federal University of Bahia, Salvador, 40155-250 Brazil; 2 Institute of Science, Technology and Innovation at the Federal University of Bahia (ICTI-UFBA), Camaçari, 42802-721 Brazil

**Keywords:** Nanophotonics and plasmonics, Nanoscience and technology, Nanoscale devices

## Abstract

Reconfigurable plasmonic-photonic electromagnetic devices have been incessantly investigated for their great ability to optically modulate through external stimuli to meet today's emerging needs, with chalcogenide phase-change materials being promising candidates due to their remarkably unique electrical and optics, enabling new perspectives in recent photonic applications. In this work, we propose a reconfigurable resonator using planar layers of stacked ultrathin films based on Metal-dielectric-PCM, which we designed and analyzed numerically by the Finite Element Method (FEM). The structure is based on thin films of Gold (Au), aluminum oxide (Al_2_O_3_), and PCM (In_3_SbTe_2_) used as substrate. The modulation between the PCM phases (amorphous and crystalline) allows the alternation from the filter to the absorber structure in the infrared (IR) spectrum (1000–2500 nm), with an efficiency greater than 70% in both cases. The influence of the thickness of the material is also analyzed to verify tolerances for manufacturing errors and dynamically control the efficiency of transmittance and absorptance peaks. The physical mechanisms of field coupling and transmitted/absorbed power density are investigated. We also analyzed the effects on polarization angles for Transversal Electric (TE) and Transversal Magnetic (TM) polarized waves for both cases.

## Introduction

The efficient control of electromagnetic waves in terahertz (THz) regions with the use of reconfigurable photonic devices is already an invaluable reality especially when it comes to metasurfaces^1–5^, metalenses^6,7^, plasmonic^8,9^ and metamaterial absorbers^10,11^. In this context, non-volatile chalcogenide phase change materials (PCM's)^12–15^ exhibit great advantages, due to their thermal stability, a guarantee of non-volatility in the drastic changes existing between the amorphous and crystalline states, ultra-fast switching between phases (nanoseconds for femtoseconds) and their optical constants values over a wide range of the electromagnetic spectrum. PCMs offer numerous technological advantages for universal memory because of their high read/write speeds, non-volatile nature, extended read/write resistance, and high scalability. An amorphous PCM film can be crystallized by heating above the crystallization temperature (or glass transition temperature), but without reaching the melting temperature. Analogously, a PCM reamorphization process involves rapidly melting and quenching the PCMs back into their amorphous phase. In a practical context, the state of materials with phase change can be controlled through temperature, and electrical voltage, among others, enabling the dynamic control of their refractive indices and, consequently, relative permittivity^16^. The high optical contrasts of materials with phase change can be perceived in the infrared spectrum, where numerous practical applications are found, such as thermal emitters^17^, camouflage^18,19^, photodetectors^20^, polarization^21^ are just some examples. The most used chalcogenide PCM’s due to their rich switchable properties are those based on Ge–Sb–Te (GST)^22–24^. GST-based PCMs have received great attention in the field of photonic reconfigurable devices and for the development of random access memory technology. Its reconfigurable characteristics and non-volatility make it possible to manipulate and control light in subwavelength geometries^25^. In recent research, the compounds of Sb_2_S_3_ and Sb_2_Se_3_, were classified as phase change materials considering their low optical losses applicable in the visible spectrum^26^. A thermally reconfigurable metasurface in the infrared region based on the GeTe phase change in^27^ and^28^ an absorber design was developed in which the phase change of the Ge–Te shifts the resonance peaks when varying partially its crystallization/amorphization. An optical and dynamically reconfigurable Metal–Insulator–Metal (MIM) filter based on Ge_2_Sb_2_Se_4_Te_1_ that can pass or attenuate near IR wavelengths was developed and tested in^29^. In^30^ the authors experimentally demonstrated two functional tuning regimes driven by the VO_2_ transition as two orders of magnitude modulation of the metasurface transmission and spectral adjustment of almost perfect absorption. Both features are accompanied by hysteresis-like behavior that can be exploited for versatile memory effects. Chen et al.^31^ proposed a VO_2_-based isotropic tunable broadband absorber in the terahertz region. Tuning the geometry at normal incidence, it was possible to achieve an absorptance efficiency greater than 90% between 1.08 and 2.55 THz.

In our study, to characterize it in a multifunctional approach, we use the InSbTe (IST) phase change into the substrate and we propose a reconfigurable device modulated by the amorphous and crystalline phases on site, which modifies the optical character of Metallic–Dielectric–Dielectric (MDD) structure high-transmission power to a Metal–Dielectric–Metal (MDM) plasmonic absorber, in infrared electromagnetic spectrum. The spatial distribution of normalized electric and magnetic fields at transmittance/absorptance peaks is also investigated. In comparison with other PCMs, IST has, despite having low optical loss in an amorphous state, after switching to the crystalline phase the real part of the permittivity is characterized by the signal change, which increases its optical loss, and the glass transition temperature is close to 300 °C while GST and GeTe is concentrated around 160 °C. Most crystalline PCM's have resonant bonds (metavalent), IST, have metallic bonds, ideal for ultrathin absorbers, optical writing and resonance control^32^. Compared to GST, IST have a higher glass transition temperature Tg (291.8 °C for IST and 160 C for GST) despite having the same melting temperatures for amorphization of the material Tm =  ~ 630 °C^33^. The phase switching of the IST is understood in the order of 10 ns for amorphization and 500 ns for crystallization^32^, while for the GST amorphization is 200 µs and 200 ns for crystallization^34^. The IST is a recently introduced phase change material and additionally presents in its crystalline phase a signal displacement of its permittivity in a broad infrared spectral band, which characterizes this phase of material as a metal. Recent investigations have been carried out as optical absorbers and resonators of plasmonic antennas^35–37^ and can, thanks to this ability, become a "plasmonic PCM", it can be used in numerous proposals as we have. Logically, the GST has unique phase change properties, which led to significant advances in the field of reconfigurable photonic devices, but in IST there are prospects for new devices for the next generation. We believe that the results demonstrated here are significant for the development of next-generation programmable optoelectronic devices.

## Device’s design

**Figure 1:**
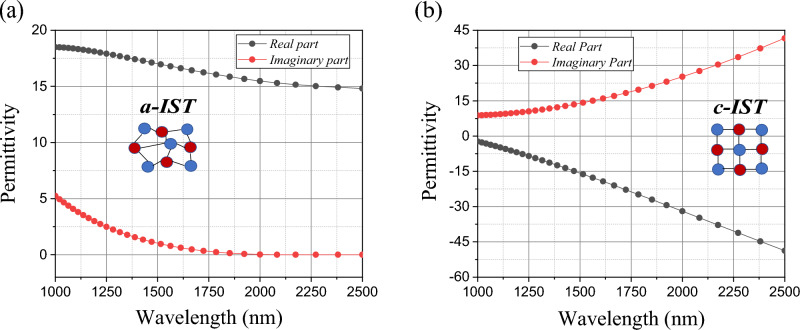
Optical constants of In_3_SbTe_2_ in the spectrum simulated in both states: (**a**) Amorphous IST; (**b**) Crystalline IST.

Our programmable and reconfigurable device is based on a planar structure, infinite in length, in three layers in which aluminum oxide (Al_2_O_3_) is sandwiched between layers of gold (Au) and phase-change material In_3_SbTe_2_. Considering the optical response, the objective of this work was to select a material with phase change as a substrate, whose function is to inhibit or filter the incident wave. The In_3_SbTe_2_ phase change material has high contrast in its relative permittivity upon crystallization, in addition to the difference between its optical losses as advantages for the simulations and the selected materials of our proposal. As the optical properties of In_3_SbTe_2_ in our work are supported by Ref.^32^, as shown in Fig. 1.

**Figure 2:**
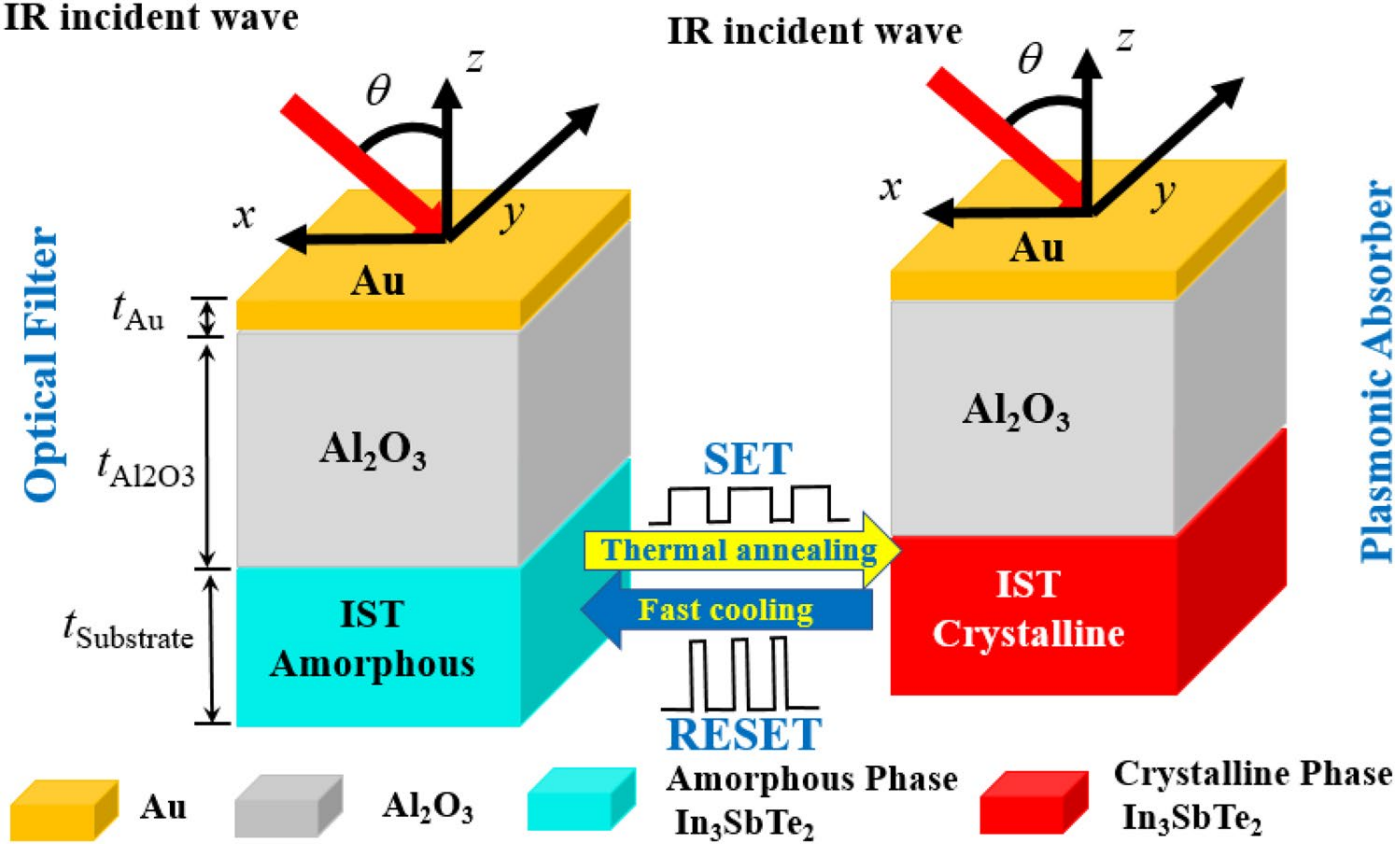
Schematic representation of the proposed model. The substrate in an amorphous state and which acts as an optical filter, upon receiving an external stimulus, switches to the crystalline phase and becomes an absorber.

Considering the relative permittivity of the materials^38^ we have that:

1 $$ \varepsilon_{r} (\omega ) = \varepsilon ^{\prime}_{r} (\omega ) - i\varepsilon ^{\prime\prime}_{r} (\omega ) $$

where $\varepsilon ^{\prime}_{r} (\omega )$ and $\varepsilon ^{\prime\prime}_{r} (\omega )$, are the real and the imaginary part of the material permittivity. From this equation and analyzing the optical constants of this material in Fig. 1a, the amorphous phase of IST has $\varepsilon ^{\prime\prime}_{r} (\omega )$ relatively low, being characterized as a semiconductor material and even as an insulator due to a $\varepsilon ^{\prime}_{r} (\omega ) > > \varepsilon ^{\prime\prime}_{r} (\omega )$. In the crystalline phase of IST, the result of negative $\varepsilon ^{\prime}_{r} (\omega )$ with positive $\varepsilon ^{\prime\prime}_{r} (\omega )$, results from a material with negative permittivity considered a metallic phase as also plasmonic for metal-dielectric interactions. Figure 2 shows the scheme of the proposed planar programmable structure of three layers, with variable thickness *t*_subst_, *t*_Al2O3_ and t_Au_, respectively. The metallic thin film of gold (Au) inserted on top of the structure, followed by the dielectric material Al_2_O_3_ and the material with phase change IST, as substrate. Crystallization of the IST occurs by long-lasting laser pulses (about 0.5 ps), with a power on the order of 10 mW, heating it above the glass transition temperature, about 291.8 °C^39^. The amorphization is obtained by heating the material with a short duration laser pulses (about 10 ns) and high power close to 300 mW, above the melting temperature (626 °C) to cool it quickly with cooling rates over 10^9^ K/s^39^.

## Numerical simulations

Numerical analyzes and simulations were performed using the frequency domain Finite Element Method^40^ in the infrared electromagnetic spectrum range (1000–2500 nm) using licensed COMSOL Multiphysics software^41^. The relative permittivity of gold ,$\varepsilon_{Au}$, is based on the Drude–Lorentz model^42^, and for the aluminum dioxide we consider $\varepsilon_{{Al_{2} O_{3} }}$ = 3.24 along the simulated and analyzed spectrum. Regarding the relative permeability of the considered materials, all of them are non-magnetic media The computational domains are 50 nm × 1331 nm in the horizontal and vertical directions. The input port is located at 1000 nm from the top of the Au layer. Full meshes consist of 1185 domain elements and 244 boundary elements for 6152 degrees of freedom., considering the initial parameters of the simulations performed *t*_Au_ = 6 nm,* t*_Al2O3_ = 295 nm and *t*_Substrate_ = 30 nm. The boundary conditions are determined from the sides of the structure and the electromagnetic wave propagates in the z direction at normal incidence and also obliquely to analyze the TE and TM modes.

Considering the incident plane wave excited at the top of the proposed structure, and with the boundary conditions periodically defined and its propagation along both *x*- and *y* directions, its light scattering magnitude is given as follows:

2 $$ A(\lambda ) + R(\lambda ) + T(\lambda ) = 1 $$

where $A(\lambda )$, $R(\lambda )$ and $T(\lambda )$, represent the absorbed, reflected, and transmitted power fraction, respectively. Specifically, here we will present the transmittance and absorptance spectra after the IST phase change analyzed in the proposed structure.

## Results and discussions

**Figure 3:**
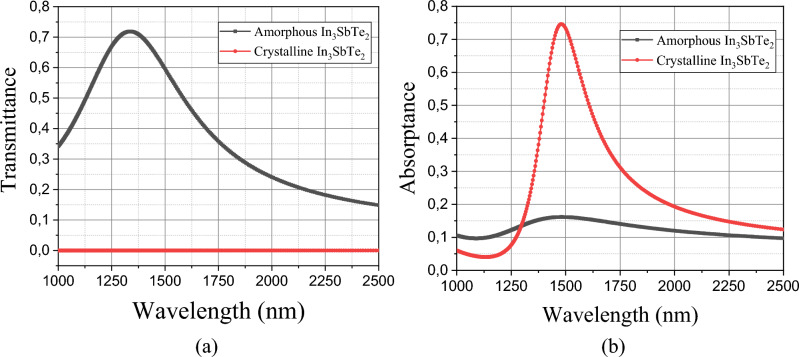
The spectrum of (**a**) Transmittance and (**b**) Absorptance of the analyzed structure with *t*_Au_ = 6 nm,* t*_Al2O3_ = 295 nm and t_Substrate_ = 30 nm.

The transmittance and absorptance spectra are shown in Fig. 3a and b. It can be seen that the high contrast between the phases switches the filter functionality for an absorber, that is, in amorphous state (a-IST), high transmittance is observed, and in the crystalline state (c-IST), high absorption happens. The used parameter were, *t*_Au_ = 6 nm,* t*_Al2O3_ = 295 nm and t_Substrate_ = 30 nm. The results reveal a transmittance peak of 72% at 1338 nm for a-IST and 0% for c-IST. Similarly, the absorptance peak of 74.8% at 1481 nm was observed for c-IST, and this value switches to 16% in a-IST.

**Figure 4:**
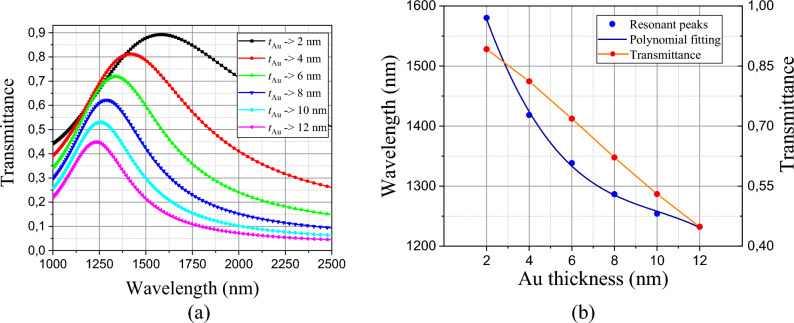
(**a**) Dependence of the transmittance spectrum with Au thickness (**b**) Polynomial fitting dependence of transmittance peaks with Au thickness.

**Figure 5:**
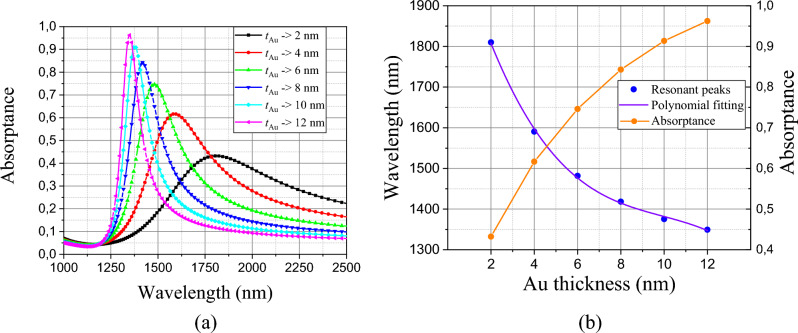
(**a**) Dependence of the absorptance spectrum with Au thickness (**b**) Polynomial fitting dependence of transmittance peaks with Au thickness.

We also numerically analyzed the efficiency of the structure, when varying the Au thickness and the numerical results can be found in Fig. 4 for the amorphous phase (filter) and in Fig. 5 for the crystalline phase (absorber). Variations were used in intervals of $\Delta t_{Au}$ = 2 nm, in order to control the resonant peak and the scattering magnitude for both cases.Fig. 4a, we can see that as the thickness increases its value, its transmittance capacity decreases proportionally and when we decrease its thickness, its transmission increases, however, due to its relatively low value, its bandwidth, defined as the band frequencies of the Full Width at Half Maximum (FWHM) becomes larger.

In Fig. 4b, we can highlight the influence of *t*_Au_ with the resonant peaks, and consequently, with its efficiency as a filter. Using empirical methods of third-degree polynomial fitting, we can control the efficiency as a filter based on the equation:

3 $$ \lambda_{res - filter} (t_{Au} ) = 1817.95 - 144.48t_{Au} + 13.27t_{Au}^{2} - 0.44t_{Au}^{3} $$

where $\lambda_{res - filter}$, is the resonant wavelength for the filter, varying from 45 to 90%, relating *t*_Au_ to its efficiency, as shown in Fig. 4b, with the most varied applications in O, E, S, and C bands window’s optical communications^43^.

Analogously to the numerical analysis performed for the structure of an amorphous substrate (filter), when changing the phase of the IST to its crystalline state (absorber), we have Fig. 5a in which the absorptance spectra were calculated. The absorber functionality increases its efficiency with the increasing golden thickness (*t*_Au_), so it decreases with decreasing golden thickness, and with relatively short FWHM compared to the filter. The design of our proposed model was also essential to achieve an absorptance range from 42 to 96% (Fig. 5b) relating resonant peaks with *t*_Au_ and which we can also control using the following equation:

4 $$ \lambda_{res - absorber} (t_{Au} ) = 2137.43 - 199.83t_{Au} + 18.72t_{Au}^{2} - 0.63t_{Au}^{3} $$

where $\lambda_{res - absorber}$, is the resonant wavelength for the filter, varying from 42 to 96%, relating *t*_Au_ to its efficiency, as shown in Fig. 5b. Gold (Au), in addition to being a highly used material in studied and published prototype absorbers, has the great advantage of considerable penetration depth, thus ensuring multifunctional efficiency, whether absorber or optical filter.

**Figure 6:**
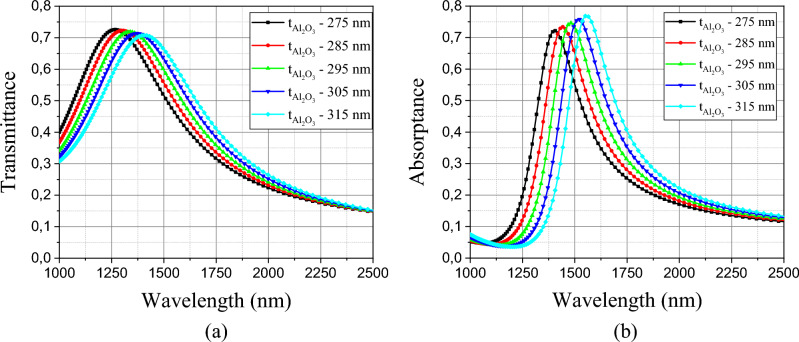
(**a**) Dependence of the transmittance spectrum with Al2O3 thickness (**b**) Dependence of the absorptance spectrum with Al_2_O_3_ thickness.

We also analyzed the influence of the aluminum oxide thickness *t*_Al2O3_ for the filter and the absorber and the results are presented in Fig. 6a and b, using a $\Delta t_{{Al_{2} O_{3} }}$ = 20 nm as interval between the ideal thickness of the model. The dimension of this thickness is relatively high and therefore there is high sensitivity when we reduce its thickness, we can shift the peak to shorter wavelengths and the opposite happens if we increase it. Its efficiency for both spectra remains above 70%, showing its great tuning capacity.

**Figure 7:**
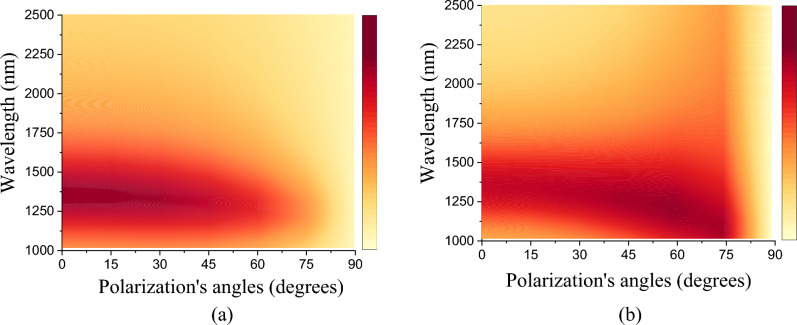
Dependence of the transmittance on the polarization and incident angles for (**a**) TE Mode and (**b**) TM Modes, respectively.

We also analyzed the dependence of polarization angles on the efficiency of our proposed model. In Fig. 7a and b it can be clearly seen that the structure has high efficiency up to an angle of 60°, regardless of the polarization mode. This means that changing the polarization and incident angle has no effect on the transmission spectra, demonstrating their angular insensitivity. Between 60° and 75° the efficiency in TE Mode starts to decrease while the TM Mode remains high and when approaching 90° they tend to decrease. Multilayer plasmonic filters with high angular insensitivity have been studied for the development of several practical applications.

**Figure 8:**
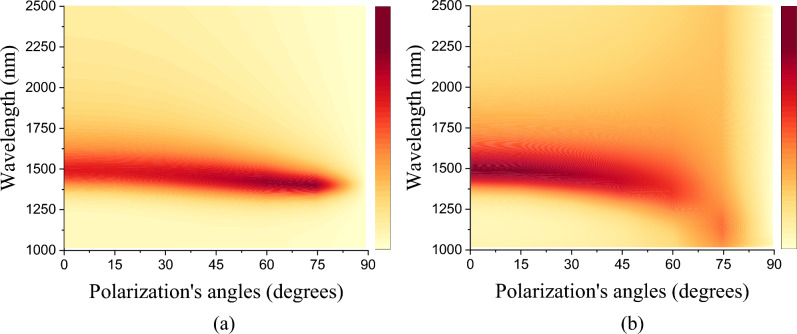
Dependence of the absorptance with the polarization and incident angles for (**a**) TE Mode and (**b**) TM Mode.

When analyzing the dependence of polarization angles on the absorptance spectrum (crystalline phase) in Fig. 8a and b, we can verify that, for the TE mode, the efficiency remains moderate along the angles and increases its intensity between 60° and 75°, in TE mode, and for the TM mode, there is a scattering and displacement of the peak for shorter wavelengths. This characteristic is directly attributed to the model's geometry and also to its multifunctional aspect when there is a switch between phases. Like filters, large angular insensitivity absorbers are essential for refractive index detectors.

**Figure 9:**
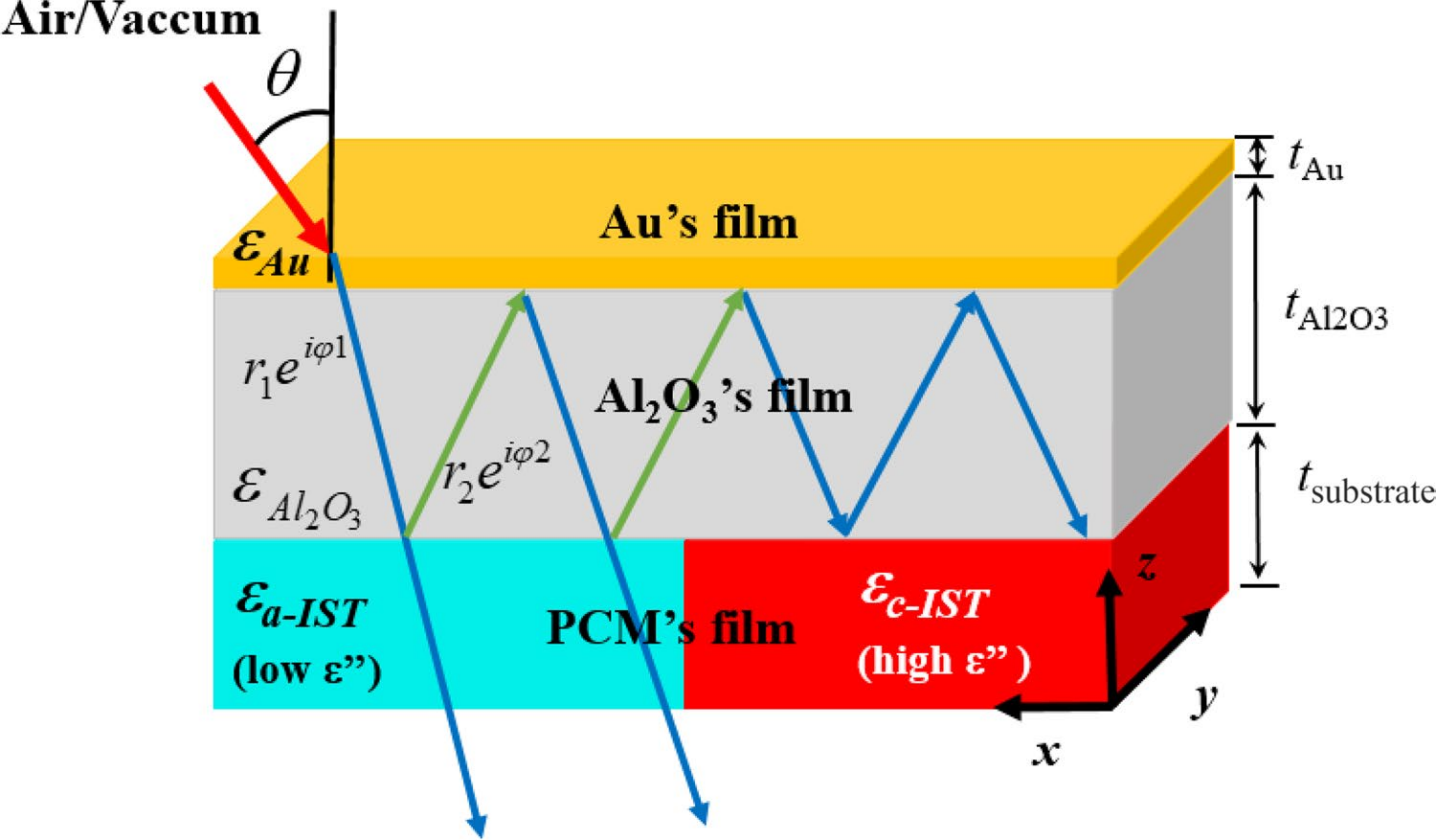
Physical coupling mechanism of structure resonant filter/absorber, incorporating a phase compensation overlap, φ1 and φ2 are the propagating phase shift at the two substrate–spacer interfaces, respectively.

Figure 9 shows the physical coupling mechanism of the hybrid multifuncional resonant structure as omnidirectional bandpass filter^44^. The geometric parameters are satisfied, and the aggregate phase shift in the PCM’s substrate/dielectric interfaces ($\varphi_{1}$ and $\varphi_{2}$) is canceled with the phase shift in the direction perpendicular to the interfaces in the dielectric region $\varphi_{1} + \varphi_{2} + 2k_{0} \sqrt {\varepsilon_{{Al_{2} O_{3} }} } t_{{Al_{2} O_{3} }} \cos \theta$^45^.The fact of using a PCM as a substrate is advantageous due to the characteristics that this specific composite provides. With the same condition satisfied, the perfect condition of constructive interference between the incident wave and the reflected/transmitted wave is given by^45^:

5 $$ \varphi_{1} + \varphi_{2} + 2k_{0} \sqrt {\varepsilon_{{Al_{2} O_{3} }} } t_{{Al_{2} O_{3} }} \cos \theta = 2m\pi $$

where $k_{0}$, is the wave vector in free space,$\sqrt {\varepsilon_{{Al_{2} O_{3} }} }$ is the Al_2_O_3_ refractive index, *t*_Al2O3_ the thickness of the layer, θ the angle of incidence and *m* = 0, ± 1, ± 2. The amorphous phase of the substrate PCM has a positive permittivity and low loss, due to its low or almost zero imaginary permittivity and superior to the resonant dielectric layer ($\varepsilon_{a - IST} > \varepsilon_{{Al_{2} O_{3} }}$). When switching to the crystalline phase, the metallic state of the substrate changes the signal and becomes a perfect absorber, due to its considerably high imaginary permittivity, common to metals ($\varepsilon_{c - IST} < \varepsilon_{{Al_{2} O_{3} }}$).

**Figure 10:**
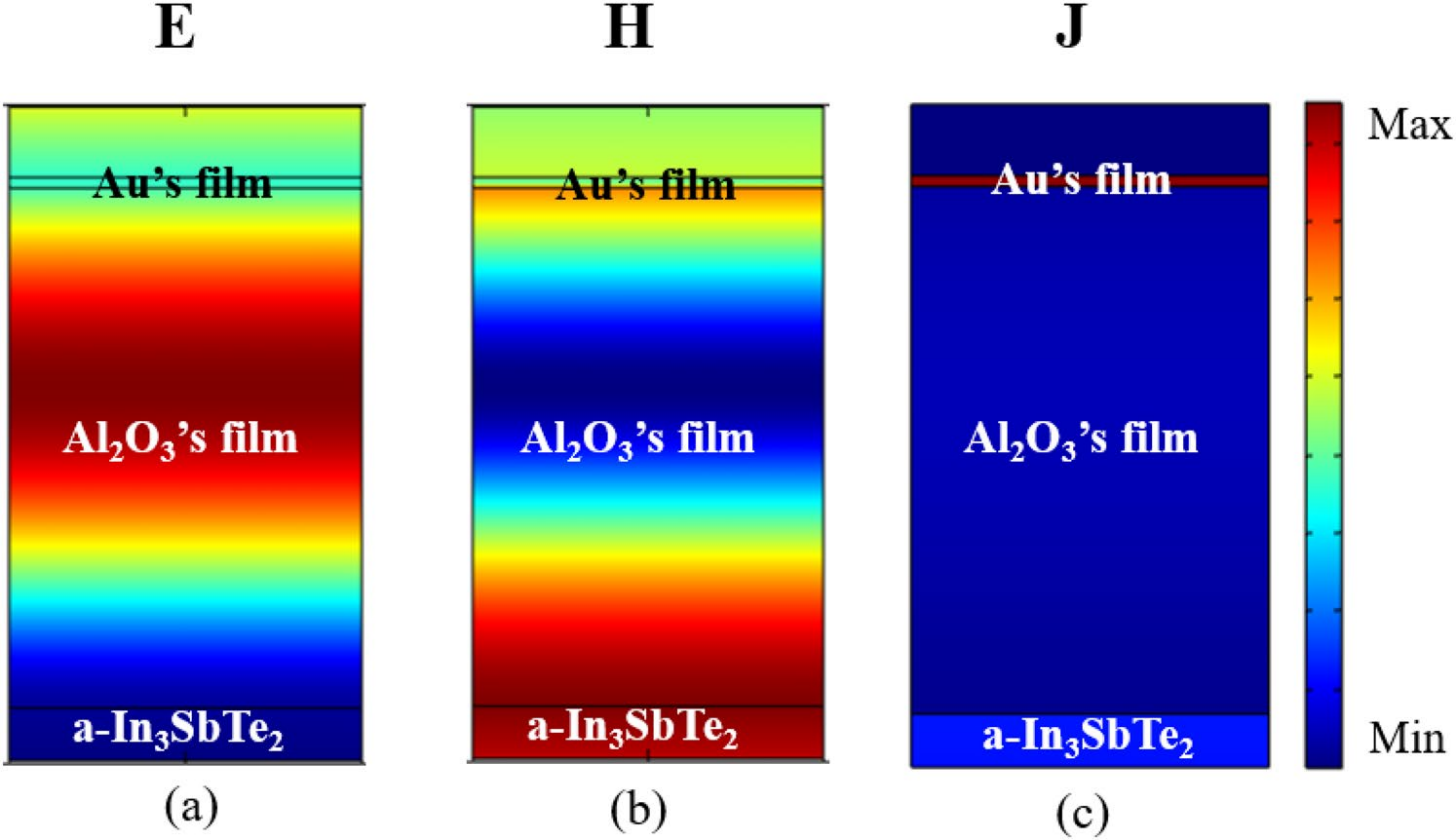
Filter’s spatial distribution of the (**a**) normalized Electric field, (**b**) normalized magnetic field, and (**c**) Current density normalized with wavelength of 1338 nm.

**Figure 11:**
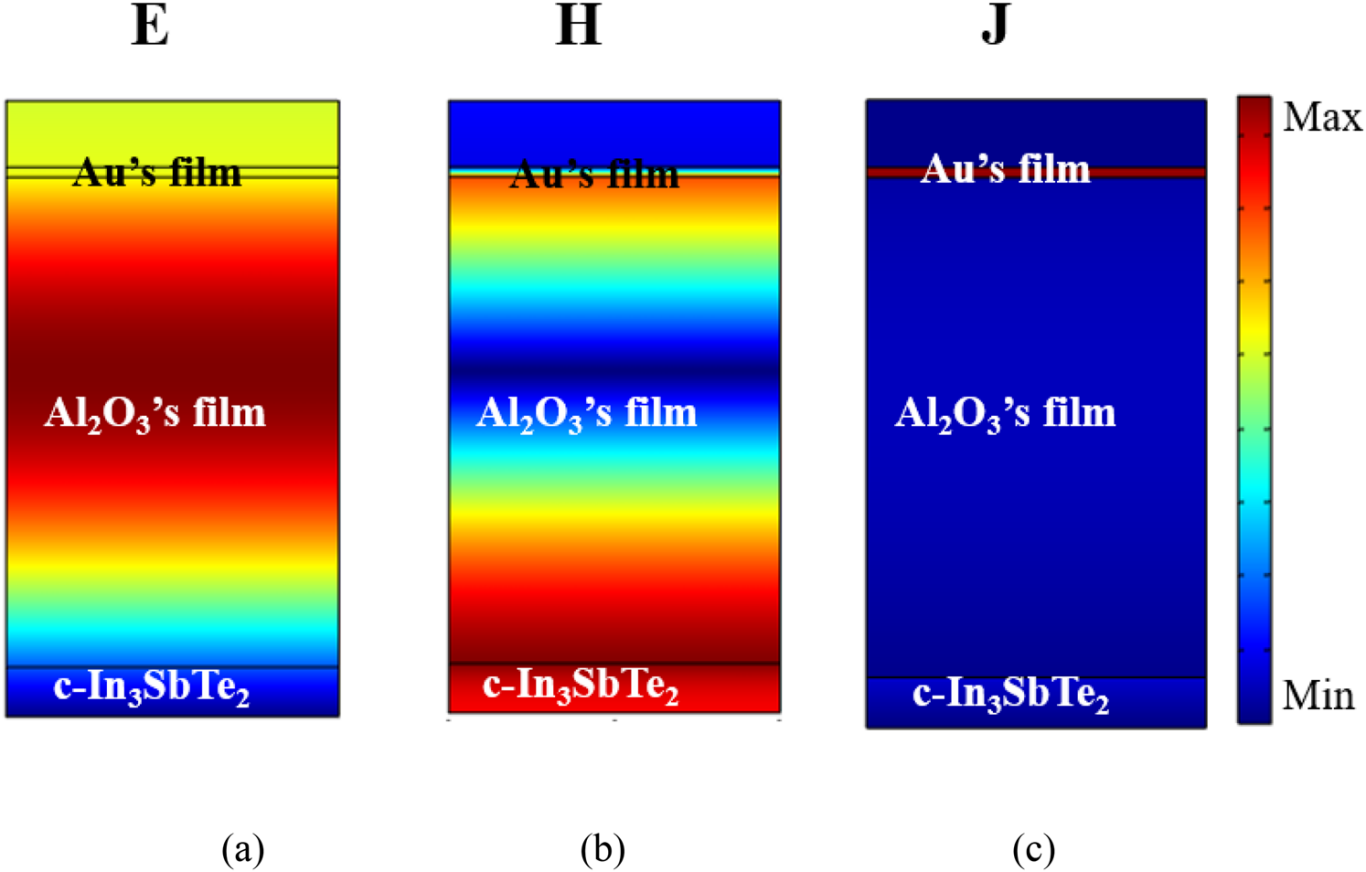
Absorber’s spatial distribution of the (**a**) normalized Electric field, (**b**) normalized magnetic field, and (**c**) Current density normalized with wavelength of 1481 nm.

To observe the physical coupling mechanism of the proposed structure to the filter and resonant absorber format, we calculated through the FEM, the normalized electric field (*E*), the normalized magnetic field (*H*), and the normalized current density distribution (*J*), considering the normal incidence and resonant peaks of λ = 1338 nm and λ = 1481 nm, for the filter (Fig. 10a–c) and for the absorber (Fig. 11a–c), respectively.

For both cases, it is possible to observe a high concentration of electric field between the Al_2_O_3_ dielectric region and the PCM substrate, caused by constructive interference in this region^28^. The result of this interference is caused by the choice of physical and geometric parameters, resulting from a high transmission using an amorphous PCM substrate (Fig. 10a) and high absorptance based on a crystalline PCM substrate (Fig. 11a). The difference that can be observed between the two cases is the resonant peaks that are in different frequencies and due to the characteristics of the structure, it has slightly higher absorptance.

Figures 10b and 11b show the spatial distribution of the normalized magnetic field **H**, where there is a strong intensity at the PCM/Al_2_O_3_ interface for both the amorphous and crystalline phases of the IST. Excitation of surface plasmon polaritons are independent of the polarization when the metallic phase of the PCM is activated. The normalized current density distribution is shown in Figs. 10c and 11c where the intense electric current acts on the upper layer of the structure, due to its metallic character and large ohmic loss.

## Conclusion

In summary, we demonstrate a programmable, tunable, controllable, and multifunctional structure using a three-layer resonator and a PCM (In_3_SbTe_2_) as a substrate. The numerically analyzed results reach an efficiency for applications such as filters or absorbers with the physical mechanism of phase change greater than 72%. We numerically demonstrate that the resonance peaks can be adjusted based on an equation, depending on the thickness of the *t*_Au_ gold layer, which can increase or decrease its efficiency and can be considered errors in the fabrication process. We can also dynamically control the resonance peaks by adjusting the thickness of the *t*_Al2O3_ dielectric spacer. The dependency of the structure with the angle of incidence was also analyzed and it was possible to note that the high transmittance/absorptance was maintained for oblique angles up to 90°. Because it is based on simple geometry, our structure allows an easy fabrication to be carried out with continuous thin film deposition. This structure may be applicable for narrow band filters/absorbers for optical communications systems and in potential applications for various technologies employed in reconfigurable nanophotonic devices, photodetectors, camouflage, refractive index sensors, and holography.

## Data Availability

All data generated or analyzed during this study are included in this article, and the datasets used or analyzed during the current study are available from the corresponding author with reasonable request.
